# Supplementary figures and images for: The DNA co-vaccination using Sm antigen and IL-10 as prophylactic experimental therapy ameliorates nephritis in a model of lupus induced by pristane
Source: PLoS One. 2021 Oct 27;16(10):e0259114. doi: 10.1371/journal.pone.0259114 (PMC8550422; doi:10.1371/journal.pone.0259114)

A

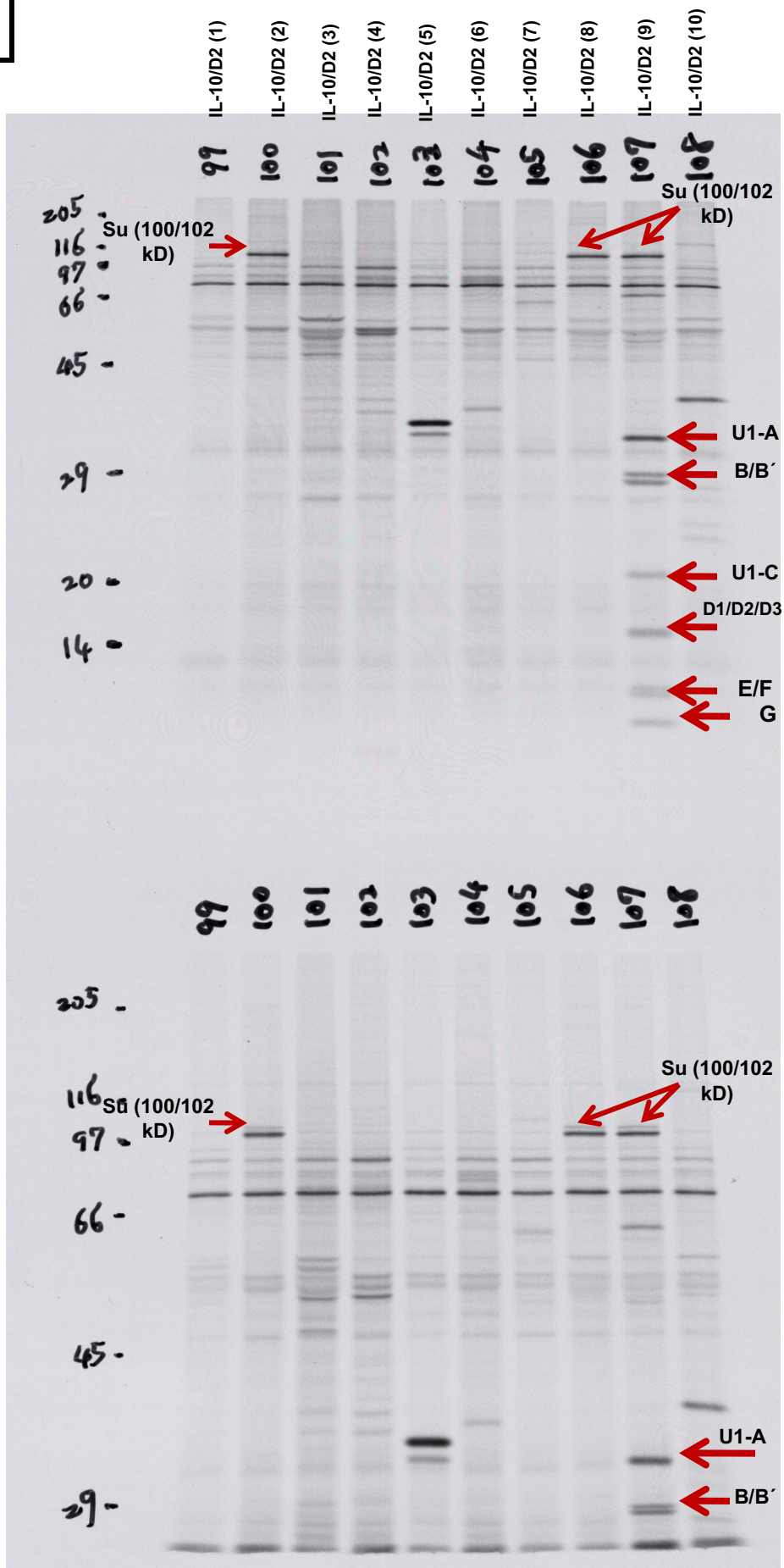

B

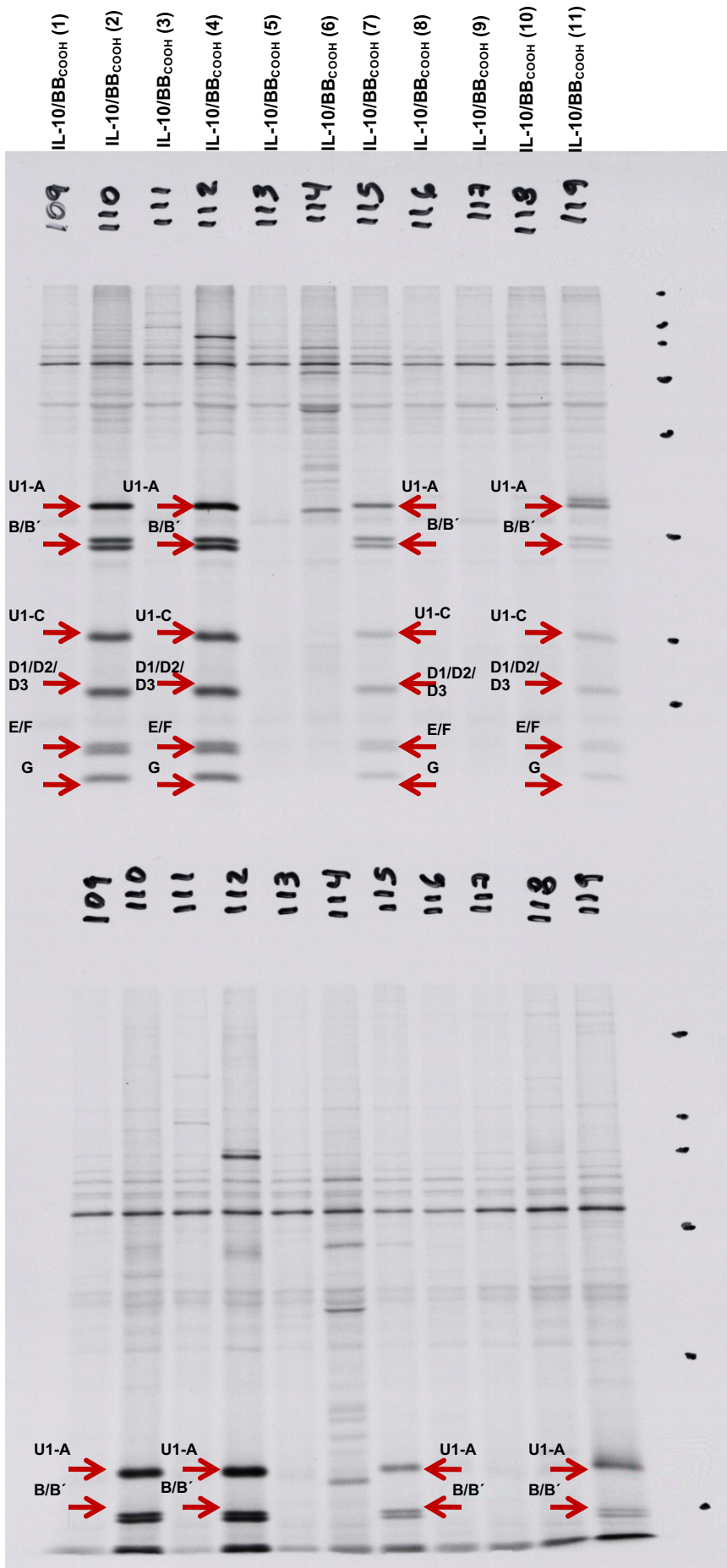

C

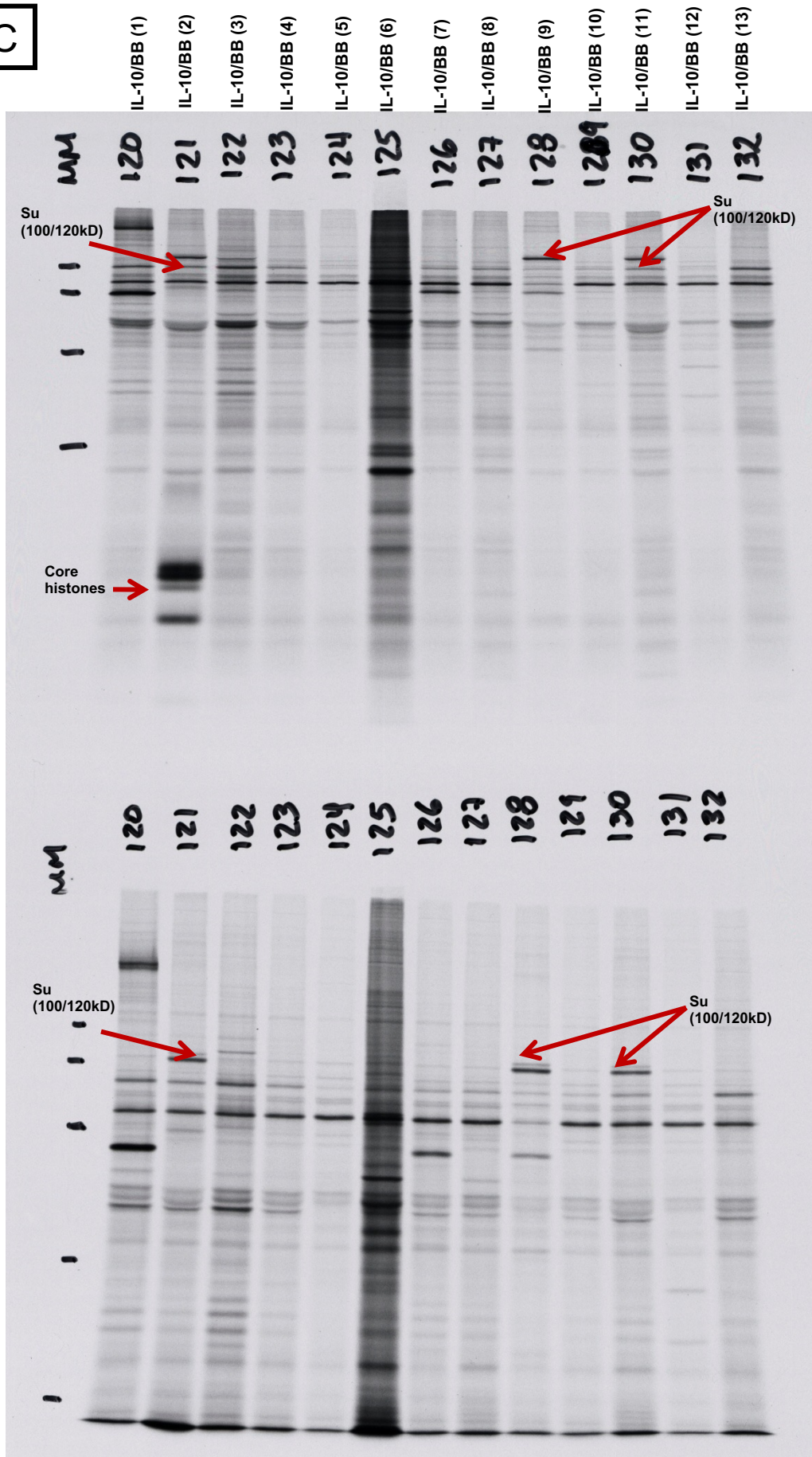

D

IL-10/D1 (1)  
IL-10/D1 (2)  
IL-10/D1 (3)  
IL-10/D1 (4)  
IL-10/D1 (5)  
IL-10/D1 (6)  
IL-10/D1 (7)  
IL-10/D1 (8)  
IL-10/D1 (9)  
IL-10/D1 (10)  
IL-10/D1 (11)  
IL-10/D1 (12)

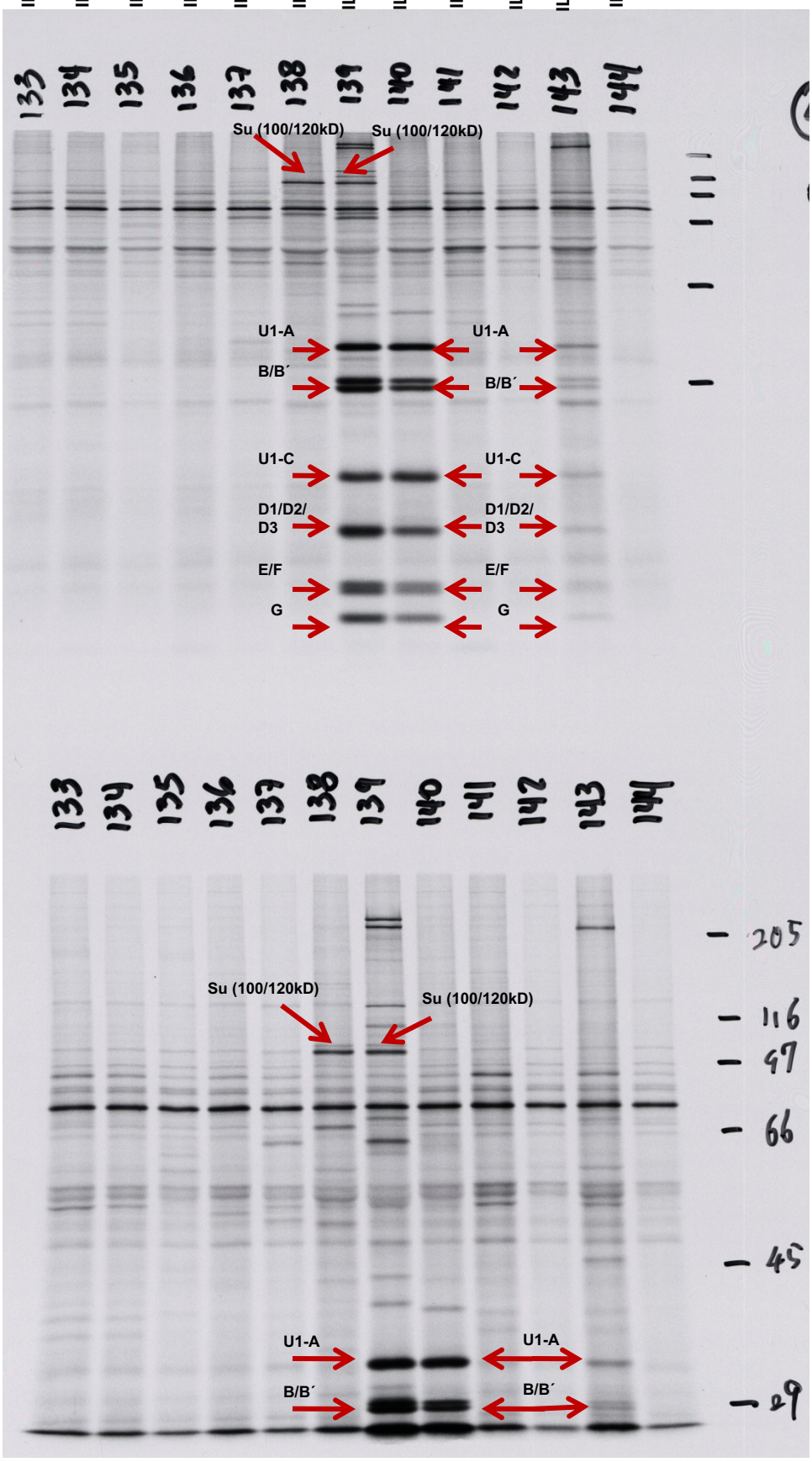

E

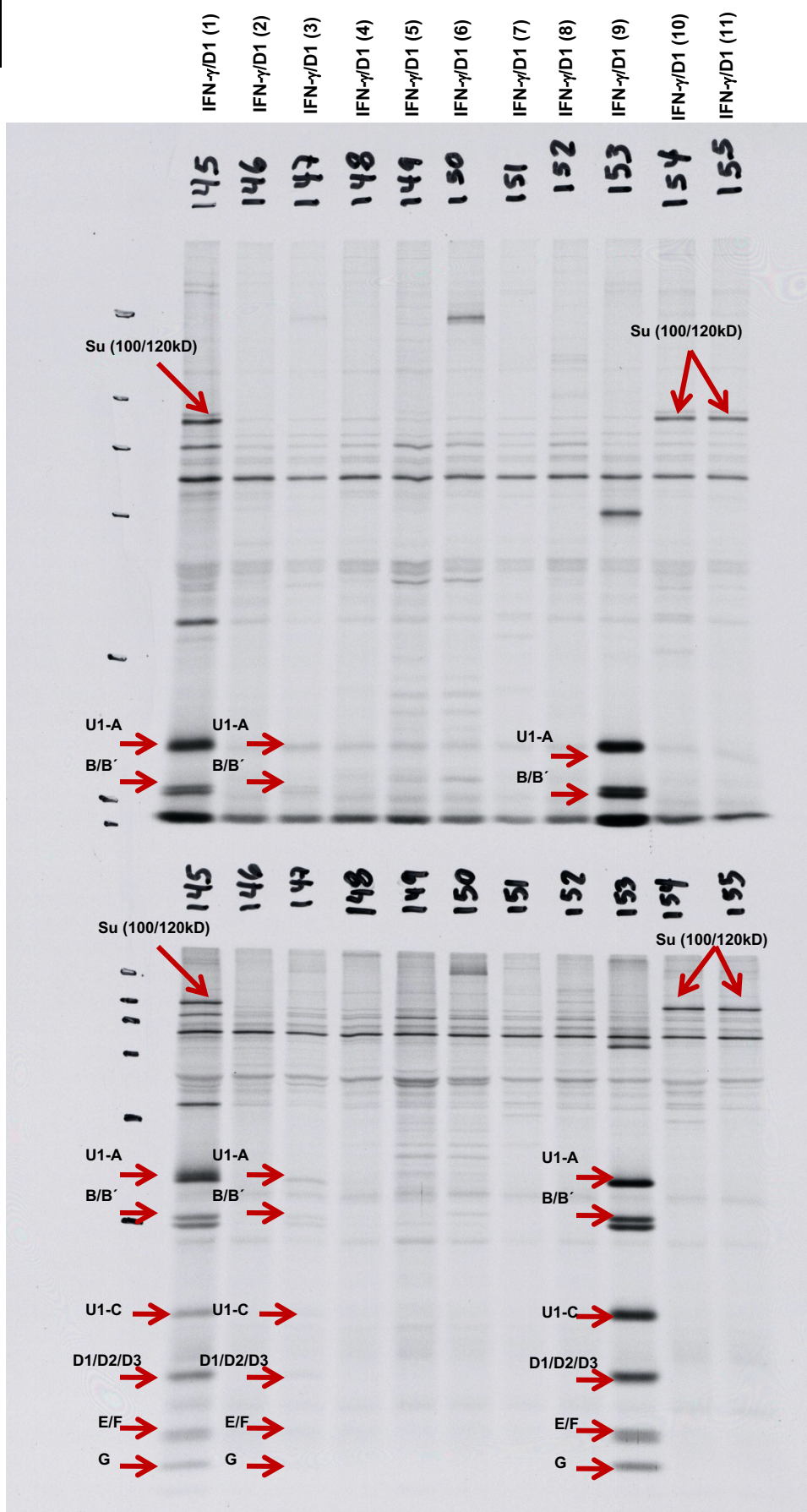

F

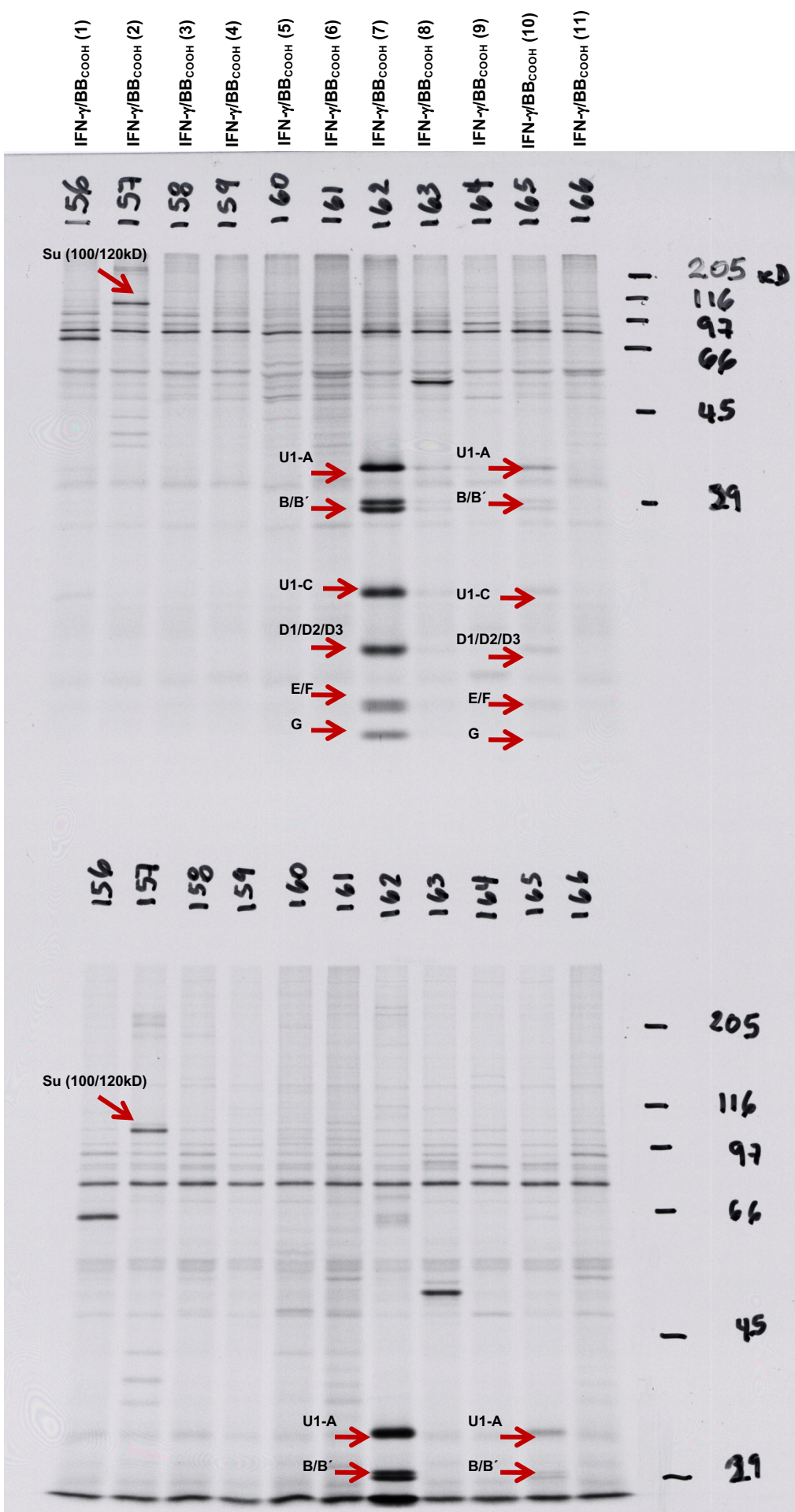

G

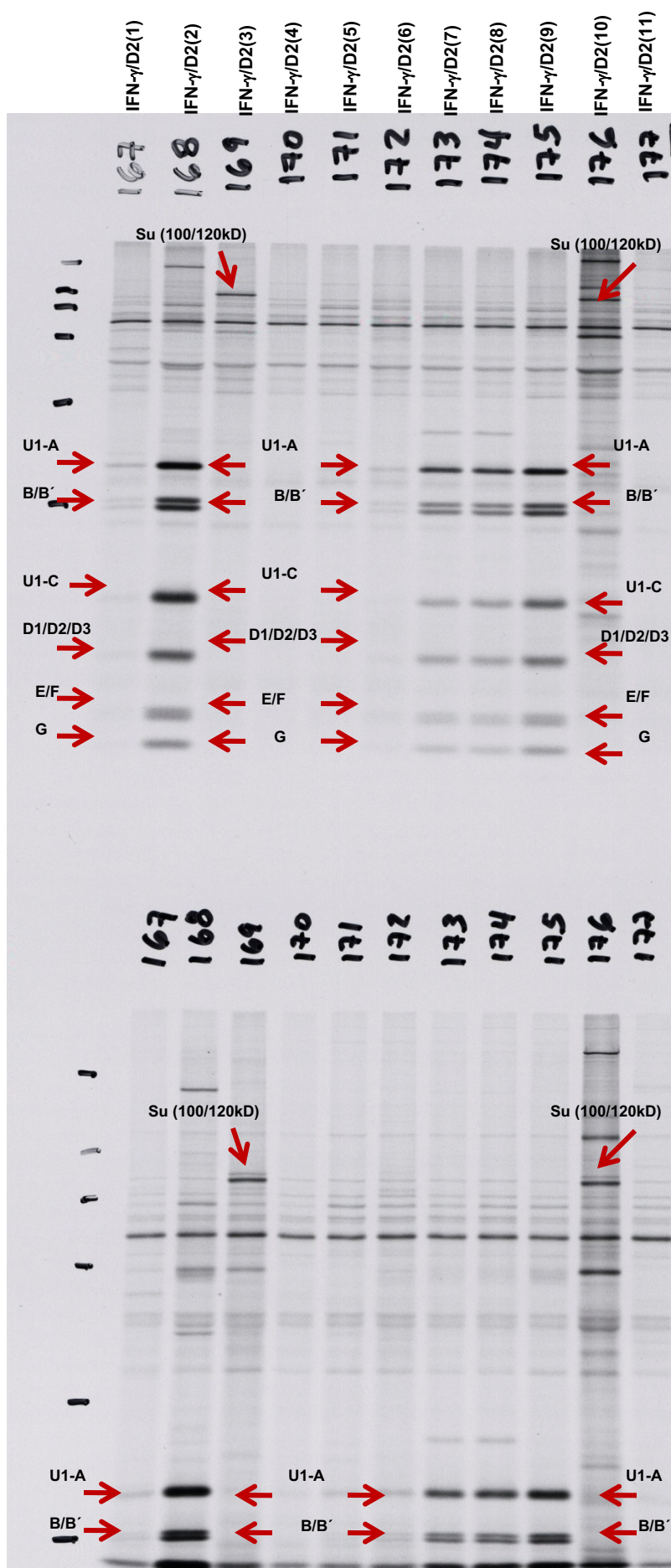

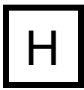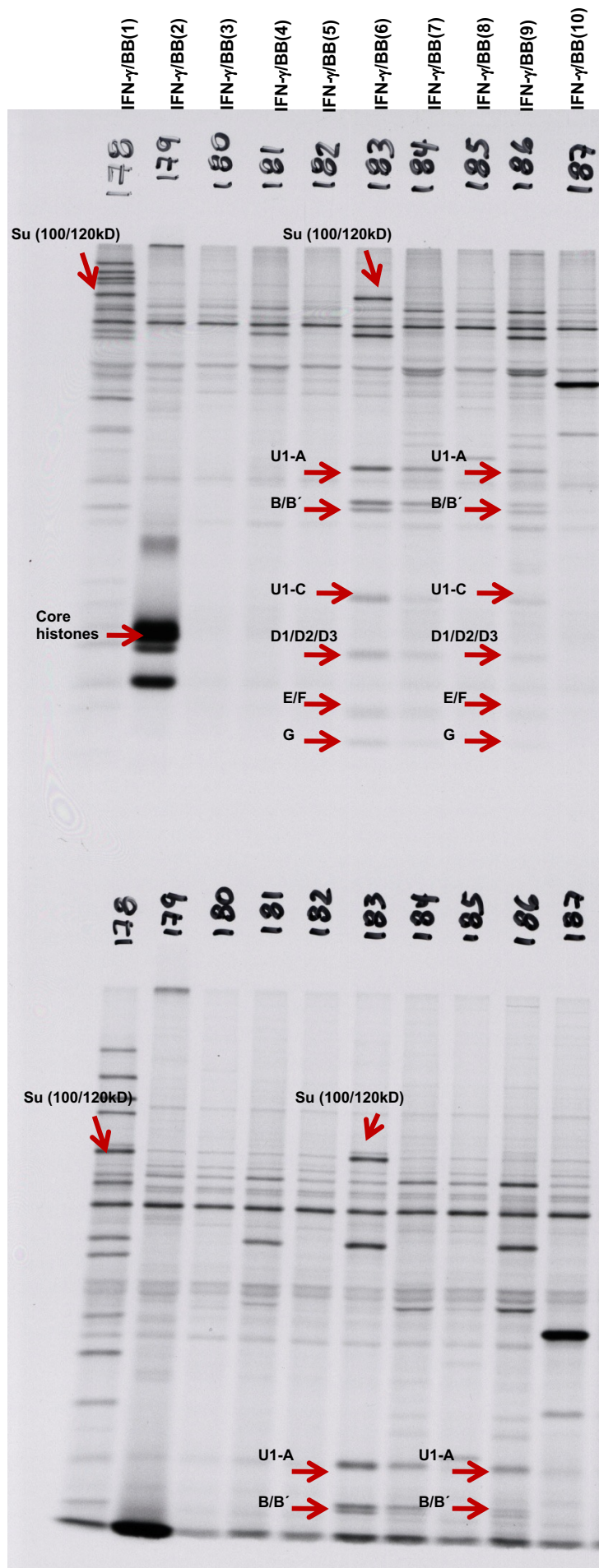

Supplement: S1 File — (PDF) [file pone.0259114.s001.pdf]

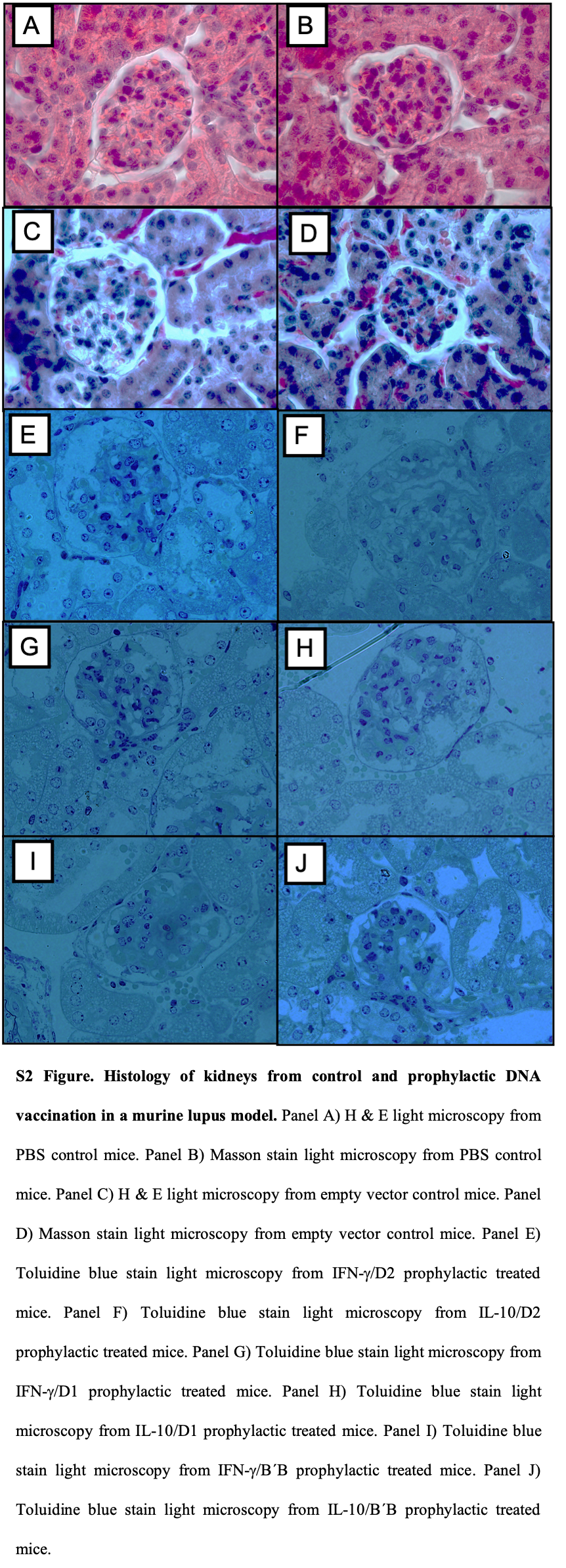

Supplement: S1 Fig — (TIF) [file pone.0259114.s003.tif]
